# The complete mitochondrial genome of a sawfly species, *Analcellicampa danfengensis* (Hymenoptera: Tenthredinidae)

**DOI:** 10.1080/23802359.2019.1666045

**Published:** 2019-09-20

**Authors:** Hongling Liu, Qingdong Chen, Yueyue Liu, Deqiang Pu, Zhiteng Chen, Xu Liu

**Affiliations:** a Key Laboratory of Integrated Pest Management of Southwest Crops, Institute of Plant Protection, Ministry of Agriculture, Sichuan Academy of Agricultural Sciences, Chengdu, China;; b Analysis and Testing Center of Sichuan Academy of Agricultural Sciences, Chengdu, Sichuan, China;; c School of Grain Science and Technology, Jiangsu University of Science and Technology, Zhenjiang, Jiangsu, China

**Keywords:** Mitochondrial genome, *Analcellicampa danfengensis*, phylogeny

## Abstract

The complete mitochondrial genome of the sawfly, *Analcellicampa danfengensis*, was sequenced and analyzed. This double strand, circular molecule is 15,968 bp in length with an A + T content of 80.9% and contains 13 PCGs, 22 tRNA genes, and two rRNA genes. Gene rearrangement occurs in the mitogenome of *A. danfengensis*. Two putative control regions are found, respectively with a length of 495 bp and 359 bp. All PCGs use standard ATN as start codons and most PCGs has complete TAN as stop codons. The phylogenetic analysis suggests that *A. danfengensis* is closely related to *Monocellicampa pruni*, another species of Tenthredinidae.

*Analcellicampa danfengensis* (Xiao 1994) (Hymenoptera: Tenthredinidae) is an economically important pest on cherry. This species is frequently found in Beijing, Hebei, Henan, Shanxi and Zhejiang provinces of China (Xiao 1994). To provide more genetic information for *A. danfengensis*, we sequenced the complete mitochondrial genome (mitogenome) of this pest for the first time and analyzed the characteristics of this biological macromolecule. A phylogenetic analysis was also performed to confirm its position in the superfamily Tenthredinoidea. The sequence is accessible in GenBank with the accession number MN163004. This study would provide important basic data for conservation genetics of *A. danfengensis* and promote the phylogenetic studies of Hymenoptera.

The male adult samples of *A. danfengensis* were collected from Sichuan Province of China (30.63 N, 104.10 E) in May 2019; all specimens and DNA are stored in the Insect Collection of Sichuan Academy of Agricultural Sciences (ICSAAS, No. ICSAAS-HYM-1), Chengdu, China. The complete mitogenome of *A. danfengensis* has a length of 15,968 bp and contains the typical set of 37 genes as in other insect, including 13 PCGs, 22 tRNA genes, two rRNA genes. There are two control regions in the mitogenome; the first one is located between *trnGln* and *trnIle*, 495 bp in length with a high A + T content of 88.5%; the second one is between *trnLeu1* and *rrnL*, 359 bp in length with a high A + T content of 91.6%. In the mitogenome of *A. danfengensis*, *trnIle-trnGln-trnMet* rearranged into *trnMet trnGln-trnIle*. The A + T content of the *A. danfengensis* mitogenome is 80.9% (A: 43.1%, T: 37.8%, C: 11.5%, and G: 7.6%), biased towards A and T nucleotides. All the PCGs start with the standard start codon ATN; most PCGs terminated with the complete stop codon TAN, but *nad4* ended with an incomplete T––. The 22 tRNA genes are varied in length from 65 bp to 71 bp, being able to fold into clover-leaf structures except for *trnSer1 (AGN)*, the DHU arm of which is completely lost. The two rRNA genes, *rrnL* and *rrnS*, are found in the conserved locations as in other insects; *rrnL* is before *trnVal*, 1364 bp in length with an A + T content of 83.0%; *rrnS* is following *trnVal*, 828 bp in length with an A + T content of 83.0%. There are 220 intergenic nucleotides dispersed between 20 gene pairs, ranging in size from 1 to 44 bp. Ten overlapped nucleotides were also found between five gene pairs; the longest overlap is between *atp8* and *atp6*.

Phylogenetic analyses for Tenthredinoidea were performed using the nucleotide sequences of 13 PCGs. The BI and ML trees generated identical topologies (Figure 1). In both trees, the monophyly of Tenthredinoidea (Tenthredinidae + Cimbicidae + Argidae) is well supported, separated from the outgroup Formicidae. In Tenthredinoidea, the three families, Tenthredinidae, Cimbicidae, and Argidae are also respectively supported as monophyletic. *Analcellicampa danfengensis* sequenced in this study is recovered as sister group of *Monocellicampa pruni*, another species from family Tenthredinidae. The phylogenetic results of this study are consistent with the results of Malm and Nyman (2015), Song et al. (2016), and Tang et al. (2019).

**Figure 1.**
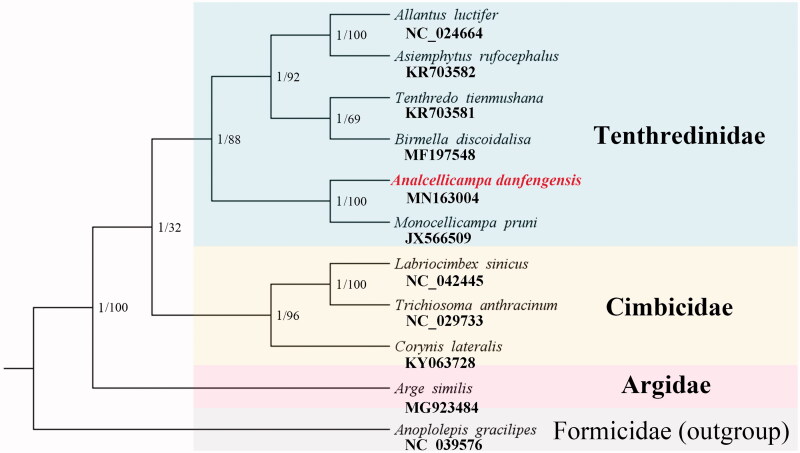
Phylogenetic tree of 11 sequenced hymenopteran species. Numbers at the nodes are posterior probabilities of BI analysis (left value) and bootstrap values of ML analysis (right value). The GenBank accession numbers are indicated under the scientific names.
